# Effects of Two Different Self-Paced Training Modalities on the Aerobic Fitness Levels, Psychophysiological Responses, and Antioxidant Status in Physically Active Young Adults

**DOI:** 10.3390/jcm12237232

**Published:** 2023-11-22

**Authors:** Yusuf Soylu, Peter Krustrup, Magni Mohr, Ersan Arslan, Bulent Kilit, Łukasz Radzimiński

**Affiliations:** 1 Faculty of Sports Sciences, Tokat Gaziosmanpasa University, Tokat 60250, Turkey; ersan.arslan@gop.edu.tr (E.A.); bulent.kilit@gop.edu.tr (B.K.); 2 Department of Sports Science and Clinical Biomechanics, SDU Sport and Health Sciences Cluster (SHSC), University of Southern Denmark, 5230 Odense, Denmark; pkrustrup@health.sdu.dk (P.K.); magnim@setur.fo (M.M.); 3 Sport and Health Sciences, College of Life and Environmental Sciences, St Luke’s Campus, University of Exeter, Exeter EX12LU, UK; 4 Danish Institute for Advanced Study—DIAS, Faculty of Health Sciences, University of Southern Denmark, 5230 Odense, Denmark; 5 Centre of Health Science, Faculty of Health, University of the Faroe Islands, 100 Tórshavn, Faroe Islands; 6 Department of Physiology and Biochemistry, Gdansk University of Physical Education and Sport, 80-336 Gdansk, Poland; lukaszradziminski@wp.pl

**Keywords:** interval training, continuous training, mood, oxidative stress, perceived exertion

## Abstract

This study aims to investigate the effects of self-paced high-intensity interval training (Sp-HIIT) vs. self-paced moderate-intensity continuous training (Sp-MICT) on aerobic fitness levels, psychophysiological responses, and antioxidant status to assess the relationship between aerobic fitness levels and antioxidant markers. Physically active young adults were randomised into Sp-HIIT and Sp-MICT groups. The intervention consisted of three weekly sessions during an eight-week period. Sp-HIIT consisted of performing two sets of 12–24 × 30 s high-intensity runs ≥ 85% HR_max_ followed by 30 s rest periods, while Sp-MICT consisted of performing 24–48 min of continuous running at 60–75% HR_max_. Pre- and post-intervention testing included a maximal oxygen uptake (VO_2max_) assessment during a 30-15 intermittent fitness test (30-15 IFT), as well as resting blood samples, which were analysed for oxidative stress markers (malondialdehyde (MDA)) and activity of intracellular antioxidant enzymes (catalase (CAT), superoxide dismutase (SOD) and reduced (GSH) and oxidized glutathione (GSSG)). The Sp-HIIT group showed a greater improvement in velocity of 30-15 IFT, VO_2max_, and MDA responses. Furthermore, the Sp-HIIT group demonstrated higher psychophysiological responses than the Sp-MICT group, except for anger responses. In conclusion, these results suggest that Sp-HIIT has a higher level of beneficial exercise-induced effects in physiological responses with greater perceived exertion in physically active young adults.

## 1. Introduction

It is widely known that physical inactivity is associated with numerous chronic diseases [1], and the World Health Organization (WHO) recommends that all adults should perform regular physical activity [2]. The positive influence of systematic exercise has a multicomponent impact on human health, including decreases in coronary heart disease risks [3] and obesity [4], support in type 2 diabetes treatment [5], and improvements in sleep quality [3], as well as mental health [6]. However, the efficiency of different types of physical activity can vary depending on volume and exercise intensity. Therefore, scientific verification of applied training programs seems to be important for sports scientists and researchers.

The modern lifestyle allows people to devote limited time to physical activity. Thus, since the beginning of the 21st century, low-volume but high-intensity interval training has drawn attention and has been explored extensively [7,8]. Repeated high-intensity training could be a time-efficient exercise mode to induce positive adaptations in skeletal muscle metabolism and upregulate aerobic capacity in men and women [9,10]. Weston et al. [11] introduced definitions of the most popular strategies of interval training based on exercise intensity. High-intensity interval training (HIIT) includes efforts at an intensity exceeding 80–85% of a person’s maximal heart rate (HR_max_), while sprint interval training (SIT) is defined as efforts at an intensity equal to or greater (supramaximal) than peak aerobic capacity. Moreover, moderate-intensity continuous training (MICT) is widely used to describe continuous efforts performed at intensities lower than HIIT [12].

The effectiveness of the training forms mentioned above has often been compared. The first comparison, published nearly half a century ago [13], reported no differences in maximal oxygen uptake (VO_2max_) changes and physiological responses between continuous and interval training participants. However, more recent studies demonstrated that interval training could provide even more benefits in physiological adaptations when training volume is matched [9]. Even when interval training volume is lower, the adaptive response is comparable or superior to endurance training [10,14,15]. Ramos et al. [16] found that HIIT enhances vascular function more effectively than MICT. Furthermore, Milanović et al. [17] observed more significant improvements in VO_2max_ following HIIT than after traditional endurance training, which is supported by Nybo et al. [15]. Similar tendencies were noted in psychological responses. It is well established that HIIT and MICT are beneficial for improving mental health [18]. Previous research has indicated that, after HIIT, participants experienced equal or greater post-exercise enjoyment than after MICT [19,20]. However, intense and extended exercise generates reactive oxygen species (ROS) and free radicals, leading to oxidative stress and potential damage to cells, proteins, lipids, and DNA [21]. Monitoring antioxidant markers is essential to mitigate oxidative stress and protect potential adverse health impacts of high-intensity exercise. Furthermore, maintaining an optimal antioxidant status can help sustain performance and reduce post-exercise muscle soreness [22].

In recent years, an expanding body of research has uncovered the influence of self-paced or self-selected intensity training on psychophysiological responses, exercise performance, and health markers in individuals at varying activity levels, including sedentary, recreationally active, and highly active levels [23,24,25]. Several recent studies have compared the effects of various training durations of HIIT and MICT interventions on adults’ psychophysiological responses, performance responses, and cardiorespiratory fitness. However, a few studies have compared the efficiency of self-paced intensities of HIIT and MICT interventions. To our knowledge, no previous study has investigated this topic in physically active young adults. Therefore, the purpose of this research was twofold: (1) to evaluate the effects of self-paced high-intensity interval training (Sp-HIIT) vs. self-paced moderate-intensity continuous training (Sp-MICT) on aerobic fitness levels, psychophysiological responses, and antioxidant status in physically active young adults; and (2) to identify potential correlations between the aerobic fitness levels and oxidative stress and antioxidant markers in this population.

## 2. Materials and Methods

### 2.1. Study Design

The effects of Sp-HIIT vs. Sp-MICT on young adults’ aerobic fitness level, psychophysiological reactions, and antioxidant status were compared using a two-group, parallel research design. Pre- and post-intervention measurements included a skinfold measurement, 30-15 intermittent fitness test (30–15 IFT), serum oxidative stress marker [malondialdehyde (MDA)], and antioxidant markers [catalase (CAT), superoxide dismutase (SOD), reduced (GSH) and oxidized glutathione (GSSG)]. Twenty-four physically active young adults were randomly assigned to either the Sp-HIIT or the Sp-MICT group. Two 12–24 × 30 s high-intensity runs at ≥85% HR_max_ were completed by the Sp-HIIT group, followed by 30 s rest periods. The participants in the Sp-MICT performed 24–48 min of continuous running at 60–75% HR_max_. These eight-week training interventions were performed three times a week, and two-day intervals separated each training session. All measurements were performed on a standard outdoor athletics track with a tartan surface between 3 and 6 o’clock in the afternoon, for similar chronobiological responses. They were told to maintain regular nutritional intake before and during the trial and were familiarised with the testing and training techniques.

Before the beginning of this study, we estimated the sample size using the G*Power software program version 3.1 (Heinrich Heine University Düsseldorf, Düsseldorf, Germany). Then, we added a partial effect size of 0.2, a power of 0.8, a *p*-value of 0.5 (2 groups and 2 number of measurements), and a correlation of 0.5, considering previous study findings of imposed vs. self-selected training [26]. We found the recommended total sample size to be 12 in this study. Twenty-four young male adults were randomly assigned to the Sp-HIIT (*n* = 12; age: 21.8 ± 1.3 years, height: 176.6 ± 5.8 cm, weight: 76.9 ± 10.6 kg) or the Sp-MICT training protocol (*n* = 12; age: 22.1 ± 1.6 years, height: 179.5 ± 5.9 cm, weight: 78.2 ± 12.2 kg). All participants were involved in team sports, including handball, basketball, and soccer, and had at least two years of experience with the training workload of four training units per week, which included core strength training, aerobic activity, and group exercise. Before this study began, written informed consent was acquired from the participants after they were informed of the research’s requirements, benefits, risks, and procedures.

### 2.2. Inclusion Criteria

- Physically active young male adults;
- Age of 20–25 years;
- Minimum of two years training (four sessions per week);
- Exclusion criteria;
- Non-attendance at training;
- Acute injuries;
- Dropped out of training.

A minimum of 10 h of fasting was allocated before their routine breakfast time (7:00–8:30 a.m.) prior to obtaining blood samples from each participant, which were collected into lithium heparin vacutainers and centrifuged at 5000× *g* rpm for 10 min at four °C to separate plasma. The plasma samples were collected and frozen at −80 °C until biochemical analysis. The spectrophotometric method was used to determine CAT [27], SOD [28] activities, and MDA levels [29]. Furthermore, GSH and GSSG concentrations were also determined spectrophotometrically, as described in a previous study [30]. After the blood collection was performed to analyse oxidative stress with MDA and antioxidant markers, the body fat percentages of participants, using the Holtain Tanner/Whitehouse Skinfold Calliper (Holtain Ltd., Crymych, UK) and Gulick Anthropometric Tape (Holtain Ltd., Crymych, UK), were estimated using the validated formula for Turkish athletes [29]. Following the anthropometric measurements, each participant performed the 30–15 IFT. This test, consisting of 30 s of running and 15 s of passive recovery, is an acoustically and reliably progressive test according to the procedures described. After the 30–15 IFT, the maximal oxygen uptake (VO_2max_) was estimated from the maximum speed (30-15 V_IFT_) reached in the last stage of the test [31].

Sp-HIIT and Sp-MICT interventions were performed on a standard athletics track three days per week for eight weeks, and two-day intervals separated each training session to maximize physical and physiological performance. A progressive training design was developed to increase final performance in both training programs. The Sp-MICT group’s total training duration was divided in half during the research period in accordance with the Sp-HIIT group. Two 12–24 × 30 s high-intensity runs at ≥85% HR_max_ were completed by the Sp-HIIT group, followed by 30 s of passive resting. The participants in the Sp-MICT group continuously performed 24–48 min of running at 60–75% HR_max_ because this type of training has been recommended for physically active young male adults to improve their physical fitness and health profiles [32,33,34]. Detailed information about training is summarized in Table 1.

The first part of each training session comprised a 15 min standardized warm-up, which included 10 min of running and 5 min of static and dynamic stretching activities. The participants were allowed to change and maintain their pace during each training session without any guidance on their activity intensity. The rating of perceived exertion (RPE) was determined using the category level (CR-20) Borg scale immediately after each session [35]. The Brunel Mood Scale (BRUMS) was used to determine mood profiles [36,37] within 10 min before and after the training sessions. The Table 1 shows the average after 3 exercise sessions per week obtained from the BRUMS. The validated and reliable scale consists of 19 items and 4 sub-scales (e.g., fatigue, depression, anger, and vigour) scored on a 5-point Likert scale [38]. Using the responses to a question of “How do you feel right now?”, participants indicated what feelings they experienced (in relation to fatigue, depression, anger, and vigour) based on the 5-point Likert scale (0 = not at all, 1 = a little, 2 = moderate, 3 = quite a bit, 4 = extreme) (Table 1).

### 2.3. Statistical Analysis

Data were represented as means ± SD. A two-factor repeated-measures analysis of variance was used to test for interactions and the main effects of time and type of group on aerobic fitness levels, psychophysiological responses, and antioxidant status. Correlations between aerobic fitness levels and pro/antioxidant markers were performed using Pearson’s (r) or Spearman’s (rho) correlation coefficient. The correlations were considered trivial (<0.1), small (0.1 to <0.3), moderate (0.3 to <0.5), large (0.5 to <0.7), very large (0.7 to <0.9), and extremely large (0.9–1.0) [39]. SPSS version 24.0 was used to conduct all statistical analyses (SPSS, Version 24.0 for Windows; SPSS Inc., Chicago, IL, USA). Statistical significance was set at the level of *p* ≤ 0.05.

## 3. Results

The weekly RPE and mood responses are demonstrated in Table 1. Overall, during the 8-week self-paced training period, the Sp-HIIT group showed higher RPE responses than those in the Sp-MICT group (*p* ≤ 0.05, d = ranging from 7.6 to 17.4). Moreover, the Sp-HIIT group also showed higher depression responses than those in the Sp-MICT group (*p* ≤ 0.05, d = ranging from 0.2 to 4.1). In contrast, the vigour responses from the Sp-MICT were significantly higher than those from the Sp-HIIT sessions (*p* ≤ 0.05, d = ranging from 0.2 to 1.3).

The Sp-HIIT and Sp-MICT interventions demonstrated similar anthropometrics, performance responses, and antioxidant status improvements except for 30-15 VFIT, VO_2max_, and MDA responses (Table 2). The Sp-HIIT showed a greater improvement in the 30-15 V_IFT_ (14.7%, *p* ≤ 0.05, d = 3.20 [very large effect]), VO_2max_ (12.2%, *p* ≤ 0.05, d = 3.15 [very large effect]), and MDA (11.2%, *p* ≤ 0.05, d = 3.59 [very large effect]) compared with the Sp-MICT group.

Table 3 demonstrates the correlations between aerobic fitness levels, oxidative stresses, and antioxidant markers during 8-week Sp-HIIT and Sp-MICT interventions. No significant relationship was found in the oxidative stresses and antioxidant markers in relation to the aerobic fitness levels of participants between pre- and post-training results.

## 4. Discussion

The main findings in our study were that both the Sp-HIIT and the Sp-MICT interventions improved body composition, aerobic fitness, and circulatory antioxidant status. However, the Sp-HIIT intervention caused greater improvements in the 30-15 V_IFT_, VO_2max_, and MDA responses, but with higher rating of perceived exertion (RPE), vigour, and depression scores, compared to Sp-MICT. Furthermore, no significant training-induced changes were found in serum oxidative stresses and antioxidant markers.

The methods for rating psychophysiological responses, such as RPE scores and mood profiles, are validated, cheap, and practical tools for measuring athletes’ physical fatigue and psychological status. Our results are consistent with the results of recent studies comparing HIIT and MICT conducted on a similar population consisting of healthy active men [40,41]. Considering the results of these studies, the HIIT intervention induced higher negative changes in mood profiles compared to the MICT. In addition to these supporting findings, the physical tiredness after a training session induced increased adverse feelings, such as anxiety, anger, and depression [42,43]. Furthermore, in a similar study, the results of RPE scores demonstrated a negative correlation with psychological responses, regardless of the participants’ physical activity levels [40]. Considering these study results, from a practical point of view, coaches and sports scientists should monitor physiological and psychological responses to observe exercise-induced implications during HIIT. The nature of HIIT and MICT might explain differences in mood state responses. While HIIT included running intervals of different durations, the MICT included prolonged exercise and had a monotonous nature, which may have affected the results. Furthermore, there could be other influencing factors, such as different types of training (imposed or self-paced exercise), participants’ training duration, and characteristics (sex, age, and type of training intervention), as previously stated [15,34].

Psychological responses to interval training were evaluated as important factors that affect exercise performance [42]. Therefore, some studies have shown an increase in negative results due to training [44,45], while others have shown an increase in positive results [46]. The HIIT intervention induced higher negative changes in mood profiles than MICT. Our results are consistent with previous studies. In dual-mode theory, HIIT may involve higher perceptual responses that override cognitive processes influencing emotional responses [44,47,48]. In addition to these supportive findings, physical fatigue after a training session increased negative emotions, such as anxiety, anger, and depression. The nature of HIIT and MICT may explain the differences in mood responses. HIIT involves running intervals of different durations, whereas MICT involves prolonged exercise and has a monotonous structure, which may have influenced cognitive processes that affect emotional responses. Nonetheless, high-intensity exercise can decrease the willingness to participate in training and pose challenges in devising exercise prescriptions.

Regarding the effect of the Sp-HIIT and Sp-MICT interventions on body composition, this study demonstrated significantly favourable effects on body weight and fat percentage. No significant differences were observed between training interventions, which confirms other findings in untrained hypertensive women [49], but contrasts findings in sedentary males [15]. However, none of these studies controlled for altered nutritional habits, which is a limitation. Furthermore, this study also demonstrated upregulated performance in the 30-15 V_IFT_ and VO_2max_ in Sp-HIIT compared to Sp-MICT after an 8-week intervention. These findings support previous research comparing HIIT and MICT interventions with different training durations ranging from 4 to 12 weeks in a variety of populations, such as healthy [32], recreationally active [13], and untrained young adults [15]. In a similar study, Hottenrott et al. [33] reported greater increases in V_IFT_ and VO_2max_ following a 12-week HIIT intervention compared with MICT in recreationally active adults. Similarly, an 8-week HIIT intervention resulted in an 11% increase in VO_2max_ in healthy men [50] and an 11% improvement in young women [51], which is of similar magnitude to that reported by Nybo et al. [15] in healthy males and by Kristiansen et al. [52] in a frail patient with coronary heart disease after 12 weeks of HIIT. In contrast to these results which support our findings, VO_2max_ has been shown to increase by 20% after a 12-week Sp-MICT training program compared with Sp-HIIT in inactive women [11]. From a practical point of view, our findings speak in favour of Sp-HIIT being a feasible, practical, and efficient training method to improve VO_2max_ in physically active adults. However, there is still no consensus on which type of self-paced training intervention is optimal for improving aerobic fitness and body composition because of the lack of literature.

This study measured circulatory oxidative stresses and antioxidant markers by determining resting serum MDA, CAT, SOD, GSH, and GSSG values prior to and after the intervention. We demonstrated a higher level of serum MDA as well as performance in the 30-15 V_FIT_ and VO_2max_ after 8 weeks of Sp-HIIT compared to Sp-MICT. In contrast to our study results, some studies have found an increase in serum SOD and CAT activity and a decrease in MDA levels in untrained men after an 8-week aerobic training intervention [53,54]. Moreover, our findings agree with Knez et al. [55], who found that the systemic MDA concentration increases progressively during this type of intervention. In addition to these findings, a recent study reported that long-term [56] and short-term [57] HIIT induced an increase in oxidative stress markers, such as MDA levels, in highly trained men. The analysis of circulatory MDA markers of peroxidation and antioxidant enzymes in individuals with long-term exposure to metabolic oxidative stresses induced by exercise training may indicate altered fatigue patterns and, consequently, the overload of adaptive stress mechanisms and depression. Although similar training intervention protocols were used in these studies, some important confounding factors, such as the population’s physical activity levels, age, and training intensity, may explain the diverging results.

Our study demonstrated that 8 weeks of Sp-HIIT provoked higher increases in 30-15 V_FIT_ and VO_2max_ responses than Sp-MICT, but showed no statistical associations between aerobic fitness levels, such as in 30-15 V_FIT_, VO_2max_ and circulatory antioxidant markers, which is in line with the findings of other studies [58]. In contrast to our study results, Peserico et al. [59] recently found strong correlations between GSSG and indicators of aerobic fitness levels, such as peak velocity and 5 km running time, in untrained men. In addition, an earlier study found that systemic GSH values were correlated with training volume and VO_2max_ in competitive triathletes [51]. Another recent study found a significant relationship between VO_2max_ and the total antioxidant status of professional soccer players during their competitive season [60]. As a result of improved VO_2max_, increased reactive oxygen species’ production is associated with increased erythrocyte glutathione peroxidase activity and glutathione concentration, both of which protect the organism against lipid peroxidation and cell membrane damage [61]. Genetic background, physical activity levels, and the general characteristics of participants may explain the differences between studies about antioxidant markers and aerobic fitness levels.

This study has strengths and limitations. The applied training modalities were designed according to the suggested physical activity guidelines to improve health conditions for adults. Furthermore, this study is the first to compare the effects of self-paced training on aerobic fitness levels, psychophysiological responses, and antioxidant status in physically active young adults. However, several limitations must be addressed. The main limitation of this study is the lack of direct measurements of aerobic capacity. Another important limitation is the lack of control over the nutritional habits of the participants, which affects physical performance in relation to body composition and blood profiles. Finally, the sample size is too small to generalize the findings, and no inactive control group was included.

## 5. Conclusions

In conclusion, both self-paced high-intensity interval and continuous moderate-intensity training in an 8-week period demonstrated similar improvements in antropometric variables and antioxidant status, while adaptations in 30-15 V_FIT_, VO_2max_, and serum MDA were in favour of high-intensity interval training. However, Sp-HIIT induced an increase in adverse feelings, such as in the RPE and depression responses, compared to Sp-MICT.

## Figures and Tables

**Table 1:** Data of the eight-week training programmes and the weekly rating of perceived exertion and fatigue, depression, anger, and vigour responses.

Weeks	Sp-MICT (*n* = 12)						Sp-HIIT (*n* = 12)					
	Training	RPE	Fatigue	Depression	Anger	Vigour	Training	RPE	Fatigue	Depression	Anger	Vigour
1	24 min cont. running	12.5 ± 0.3	2.4 ± 0.4	1.7 ± 1.0	1.6 ± 0.8	1.4 ± 0.4 *	2 × (12 × 30 s), 30 s rest	16.2 ± 0.2 *	3.0 ± 0.4	3.2 ± 1.3 *	1.6 ± 1.0	0.9 ± 0.4
2		13.2 ± 0.3	2.9 ± 0.5	1.6 ± 0.5	1.6 ± 0.2	1.1 ± 0.4 *		17.1 ± 0.1 *	3.3 ± 0.4	2.3 ± 0.9 *	1.3 ± 0.4	0.7 ± 0.4
3	32 min cont. running	13.1 ± 0.5	3.0 ± 0.4	1.6 ± 0.5	1.3 ± 0.2	0.9 ± 0.4 *	2 × (16 × 30 s), 30 s rest	17.1 ± 0.2 *	3.3 ± 0.3	2.0 ± 0.6 *	1.3 ± 0.3	0.6 ± 0.3
4		14.1 ± 0.4	2.9 ± 0.6	1.1 ± 0.6	1.1 ± 0.4	1.1 ± 0.6 *		17.3 ± 0.1 *	3.2 ± 0.4	2.1 ± 0.6 *	1.1 ± 0.5	1.8 ± 0.4
5	40 min cont. running	13.6 ± 0.7	2.8 ± 0.4	1.4 ± 0.6	1.1 ± 0.5	1.2 ± 0.4 *	2 × (20 × 30 s), 30 s rest	17.4 ± 0.1 *	3.1 ± 0.3	2.2 ± 0.6 *	0.9 ± 0.4	0.9 ± 0.3
6		13.4 ± 0.1	2.8 ± 0.5	0.9 ± 0.5	1.1 ± 0.4	0.9 ± 0.4 *		17.8 ± 0.4 *	3.2 ± 0.3	1.3 ± 0.4 *	0.9 ± 0.3	0.7 ± 0.3
7	48 min cont. running	13.6 ± 0.3	3.9 ± 0.5	0.8 ± 0.4	1.1 ± 0.5	1.1 ± 0.5 *	2 × (24 × 30 s), 30 s rest	17.7 ± 0.3 *	3.3 ± 0.3	0.9 ± 0.5 *	0.8 ± 0.4	0.7 ± 0.3
8		13.7 ± 0.6	2.9 ± 0.6	0.8 ± 0.7	0.9 ± 0.6	1.0 ± 0.5 *		17.9 ± 0.3 *	3.4 ± 0.3	1.0 ± 0.6 *	0.8 ± 0.4	0.6 ± 0.3

Sp-HIIT: high-intensity interval training; Sp-MICT: moderate-intensity continuous training; RPE: rating of perceived exertion; * *p* ≤ 0.05 for between-group changes.

**Table 2:** Effect of 8 weeks of training on anthropometrics, aerobic fitness levels, and antioxidant status of the participants.

	Sp-MICT (*n* = 12)				Sp-HIIT (*n* = 12)				*p* Values
	Pre-Test	Post-Test	Cohen’s *d*	Descriptor	Pre-Test	Post-Test	Cohen’s *d*	Descriptor	
Body weight (kg)	78.2 ± 12.2	76.1 ± 11.8 *	0.2	trivial	75.7 ± 8.9	73.2 ± 8.6 *	0.3	small	0.540
Body fat (%)	14.8 ± 3.7	13.2 ± 3.2 *	0.4	small	13.9 ± 3.0	11.8 ± 2.4 *	0.8	moderate	0.365
BMI (kg·m^−2^)	24.2 ± 2.9	23.5 ± 2.8 *	0.2	small	25.0 ± 2.4	24.2 ± 2.3 *	0.3	small	0.462
30-15 V_IFT_ (km·h^−1^)	14.5 ± 0.5	15.1 ± 0.6 *	1.0	moderate	14.3 ± 0.6	16.4 ± 0.7 *#	3.2	very large	0.040
VO_2max_ (mL·min^−1^·kg^−1^)	40.7 ± 1.3	42.1 ± 1.5 *	1.1	moderate	40.2 ± 1.5	45.1 ± 1.6 *#	3.1	very large	0.043
MDA (mmol.ml^−1^)	0.4 ± 0.0	0.4 ± 0.0 *	2.0	very large	0.4 ± 0.0	0.5 ± 0.0 *#	3.2	very large	0.025
CAT (U·mg^−1^ Hb)	66.9 ± 11.1	52.0 ± 11.5 *	1.3	large	64.5 ± 6.3	44.8 ± 4.5 *	3.6	very large	0.191
SOD (U·mg^−1^ Hb)	1.4 ± 0.2	1.3 ± 0.1 *	0.9	moderate	1.4 ± 0.2	1.2 ± 0.2 *	1.5	large	0.309
GSH (µmol·g^−1^ Hb)	11.4 ± 0.7	13.5 ± 0.6 *	3.3	very large	11.7 ± 0.7	14.1 ± 1.2 *	2.4	very large	0.208
GSSG (µmol·g^−1^ Hb)	8.9 ± 1.0	10.5 ± 1.1 *	1.5	large	9.4 ± 0.7	10.9 ± 0.7 *	2.1	very large	0.226
GSH/GSSG ratio	1.3 ± 0.1	1.3 ± 0.1 *	0.1	trivial	1.2 ± 0.1	1.3 ± 0.2 *	0.5	small	0.755

Data presented as means ± SD. BMI: body mass index; 30-15 V_IFT_: maximum speed reached in the last stage of the 30-15 intermittent fitness test; VO_2max_: maximal oxygen uptake; CAT: catalase; SOD: superoxide dismutase; MDA: malondialdehyde; GSH: glutathione; GSSG: oxidized glutathione. * *p* ≤ 0.05 for within-group changes. # *p* ≤ 0.05 for between-group changes.

**Table 3:** Correlations between aerobic fitness levels and oxidative stresses and antioxidant markers.

	CAT (U·mg^−1^ Hb)		SOD (U·mg^−1^ Hb)		MDA (mmol·mL^−1^)		GSH (µmol·g^−1^ Hb)		GSSG (µmol·g^−1^ Hb)		GSH/GSSG Ratio	
	Pre	Post	Pre	Post	Pre	Post	Pre	Post	Pre	Post	Pre	Post
30-15 V_IFT_ (km·h^−1^)	−0.1	−0.1	−0.2	−0.2	−0.2	−0.1	−0.2	−0.3	−0.1	−0.3	0.1	0.2
	(small)	(trivial)	(small)	(small)	(small)	(small)	(small)	(small)	(small)	(moderate)	(trivial)	(small)
VO_2max_ (mL·min^−1^·kg^−1^)	0.0	0.0	0.2	0.1	0.1	0.1	0.1	0.2	0.1	0.4	−0.1	−0.3
	(trivial)	(trivial)	(small)	(small)	(small)	(small)	(small)	(small)	(trivial)	(moderate)	(trivial)	(small)

Data presented as means ± SD. 30-15 V_IFT_: maximum speed reached in the last stage of the 30-15 intermittent fitness test; VO_2max_: maximal oxygen uptake; CAT: catalase; SOD: superoxide dismutase; MDA: malondialdehyde; GSH: glutathione; GSSG: oxidized glutathione.

## Data Availability

The original contributions generated for this study are included in the article; further inquiries can be directed to the corresponding author.
